# Identification, cloning and characterization of SpEX exotoxin produced by *Staphylococcus pseudintermedius*

**DOI:** 10.1371/journal.pone.0220301

**Published:** 2019-07-29

**Authors:** Mohamed A. Abouelkhair, David A. Bemis, Richard J. Giannone, Linda A. Frank, Stephen A. Kania

**Affiliations:** 1 Department of Biomedical and Diagnostic Sciences, University of Tennessee College of Veterinary Medicine, Knoxville, Tennessee, United States of America; 2 Department of Bacteriology, Mycology and Immunology, Faculty of Veterinary Medicine, University of Sadat City, Menoufia, Egypt; 3 Chemical Sciences Division, Biological Mass Spectrometry, Oak Ridge National Laboratory, Oak Ridge, Tennessee, United States of America; 4 Department of Small Animal Clinical Sciences, College of Veterinary Medicine, University of Tennessee, Knoxville, TN, United States of America; Indiana University School of Medicine-Northwest, UNITED STATES

## Abstract

*Staphylococci* have evolved numerous strategies to evade their hosts’ immune systems. Some staphylococcal toxins target essential components of host innate immunity, one of the two main branches of the immune system. Analysis of the *Staphylococcus pseudintermedius* secretome using liquid chromatography mass spectrometry guided by genomic data, was used to identify an *S*. *pseudintermedius* exotoxin provisionally named SpEX. This exoprotein has low overall amino acid identity with the *Staphylococcus aureus* group of proteins named staphylococcal superantigen like proteins (SSLs) and staphylococcal enterotoxin- like toxin X (SEIX), but predictive modeling showed that it shares similar folds and domain architecture to these important virulence factors. In this study, we found SpEX binds to complement component C5, prevents complement mediated lysis of sensitized bovine red blood cells, kills polymorphonuclear leukocytes and monocytes and inhibits neutrophil migration at sub-lethal concentrations. A mutant version of SpEX, produced through amino acid substitution at selected positions, had diminished cytotoxicity. Anti-SpEX produced in dogs reduced the inhibitory effect of native SpEX on canine neutrophil migration and protected immune cells from the toxic effects of the native recombinant protein. These results suggest that SpEX likely plays an important role in *S*. *pseudintermedius* virulence and that attenuated SpEX may be an important candidate for inclusion in a vaccine against *S*. *pseudintermedius* infections.

## Introduction

*Staphylococcus pseudintermedius* is the main cause of canine dermatological disease and has been isolated from wound and surgical site infections, endocarditis and mastitis in dogs [1, 2]. Human infections with this organism have been reported sporadically, most of which have been related to exposure to dogs [3]. As many as 30–40% of the *S*. *pseudintermedius* isolates tested in clinical laboratories in different geographical areas are methicillin-resistant (MRSP) [4]. Most MRSP are multidrug-resistant leaving few treatment options. The transfer of resistance genes to human organisms as well as direct transfer of multidrug-resistant *S*. *pseudintermedius* between animals and to humans are theoretical concerns [5–8]. Development of alternative approaches to control staphylococcal infections, such as vaccines, is challenging. *S*. *pseudintermedius* may evade their hosts’ immune response by producing a number of cell surface and secreted proteins that target essential components of their hosts’ defenses, promoting the survival and transmission of this pathogen. *S*. *pseudintermedius*, like *Staphylococcus aureus*, produces hemolysins, exfoliative toxins, leukotoxins, thermonuclease, protein A, coagulase and adenosine synthase [4, 9–13]. However, many of these virulence factors are not well characterized in *S*. *pseudintermedius* and others likely remain to be identified.

Innate immunity involving neutrophils is an important first step in the defense against staphylococci and migration from blood provides the majority of these effectors. This process is triggered by inflammatory signal molecules such as C5a, neutrophils bind to upregulated endothelial selectins, extravasate and migrate toward the site of inflammation [14]. However, neutrophils are susceptible to staphylococcal defenses including molecules that inhibit neutrophil function and leukotoxins that kill by forming pores in their cell membranes [15].

The roles of structurally related *S*. *aureus* secreted virulence factors staphylococcal superantigen-like proteins (SSLs) and staphylococcal enterotoxin-like toxin (SEIX) in inhibiting their host’s innate immune response are well characterized. SSLs are distinguished from SEIX by their lack of cytotoxic activity. SSLs are two- domain proteins with an average size of 25 kDa [16, 17]. The first domain, located at the N-terminus, displays an oligosaccharide/ oligonucleotide binding (OB) fold forming a β- barrel. The second domain possesses a β-grasp motif consisting of a twisted β-sheet of four to five antiparallel strands, located at the C-terminus. The two domains are separated by a structurally conserved α-helix. SSLs target innate immunity components but do not bind to T cell receptors or the major histocompatibility complex [18, 19].

In previous studies, *S*. *aureus* SSL4 [19], SSL5 [17] and SSL11 [17], were found to interfere with neutrophil migration through their sialated glycan-binding site in the C- terminal β-grasp domain. Chung et al., [20] showed that SSL11 with a single site mutation, T168P, had defective binding compared to native SSL11 and lost its inhibitory effect on neutrophil attachment to P-selectin. The OB fold domain of SSL7 binds to the IgA Fc region while the β-grasp motif adheres tightly to complement component C5 resulting in inhibition of complement mediated hemolytic activity [17]. SSL3 binds to and inhibits toll-like receptor (TLR) 2 using a well characterized recognition site not found in other SSLs [21]. Tuffs et al., [22] showed that SEIX secreted by *S*. *aureus* shares its domain structure with SSLs. As with SSLs, SEIX binds to neutrophils, however, it also exhibits a superantigenic effect via its OB fold on T cells [19, 22].

In this study, we used a proteomic approach to identify an exoprotein produced by *S*. *pseudintermedius*, SpEX. It is most closely related to and shares some biological properties and domain structures with SSLs and SEIX, however, it has less than 50% amino acid similarity with SSL proteins and has key distinguishing properties. It is often annotated in *S*. *pseudintermedius* as exotoxin 15, a synonym for SSL11, formerly referred to as staphylococcal exotoxin-like proteins (SET) [23]. SpEX was studied to determine if it had immunosuppressive effects on its host’s innate immune system. The specific objectives of this study were to characterize the immunobiological properties of *S*. *pseudintermedius* SpEX and attenuated (reduced toxicity) SpEX (SpEX-M) by measuring their inhibitory effects on complement activity, neutrophil migration and cytotoxic effects on PMNs and monocytes. Antibody against SpEX-M was developed in clinically healthy dogs and the neutralizing activity of those antibodies was evaluated.

## Results

### Identification of putative immune modulator from *S*. *pseudintermedius*

Liquid chromatography–mass spectrometry (LC-MS/MS) was used to screen culture supernatants of three clinical strains of *S*. *pseudintermedius* (06–3228, 08–1661 and NA45), representing the major *S*. *pseudintermedius* genotypes occurring in the United States as determined by multilocus sequence typing (8). We identified over 500 secreted or cell wall associated proteins and others with unknown cellular locations (manuscript under review). The secretome proteins were compared among 06–3228, 08–1661 and NA45 strains using their respective genomes as references. SpEX, a 234-aa protein, was detected in the supernatants of the three isolates. The mean predicted molecular weight was 26.093 kDa with a mean isoelectric point of 6.30.

### Bioinformatics analysis of *S*. *pseudintermedius* SpEX

A BLAST search of SpEX in the GenBank database revealed that it is conserved among *S*. *pseudintermedius* strains including 06–3221, 08–1661 and NA45 with amino acid identities over 88% between strains. SpEX shares sequence similarity with other pathogenic bacterial species within the *Staphylococcus intermedius* group. It shares amino acid identity of 71.8% with *Staphylococcus intermedius*, 73.5% with *Staphylococcus delphini* and 72.2% with *Staphylococcus cornubiensis*. No similar proteins were identified in *S*. *aureus*. SpEX has approximately 47% amino acid sequence identity with *S*. *aureus* SSL11 and less than 30% amino acid identity with other SSL members and SEIX.

**Fig 1:**
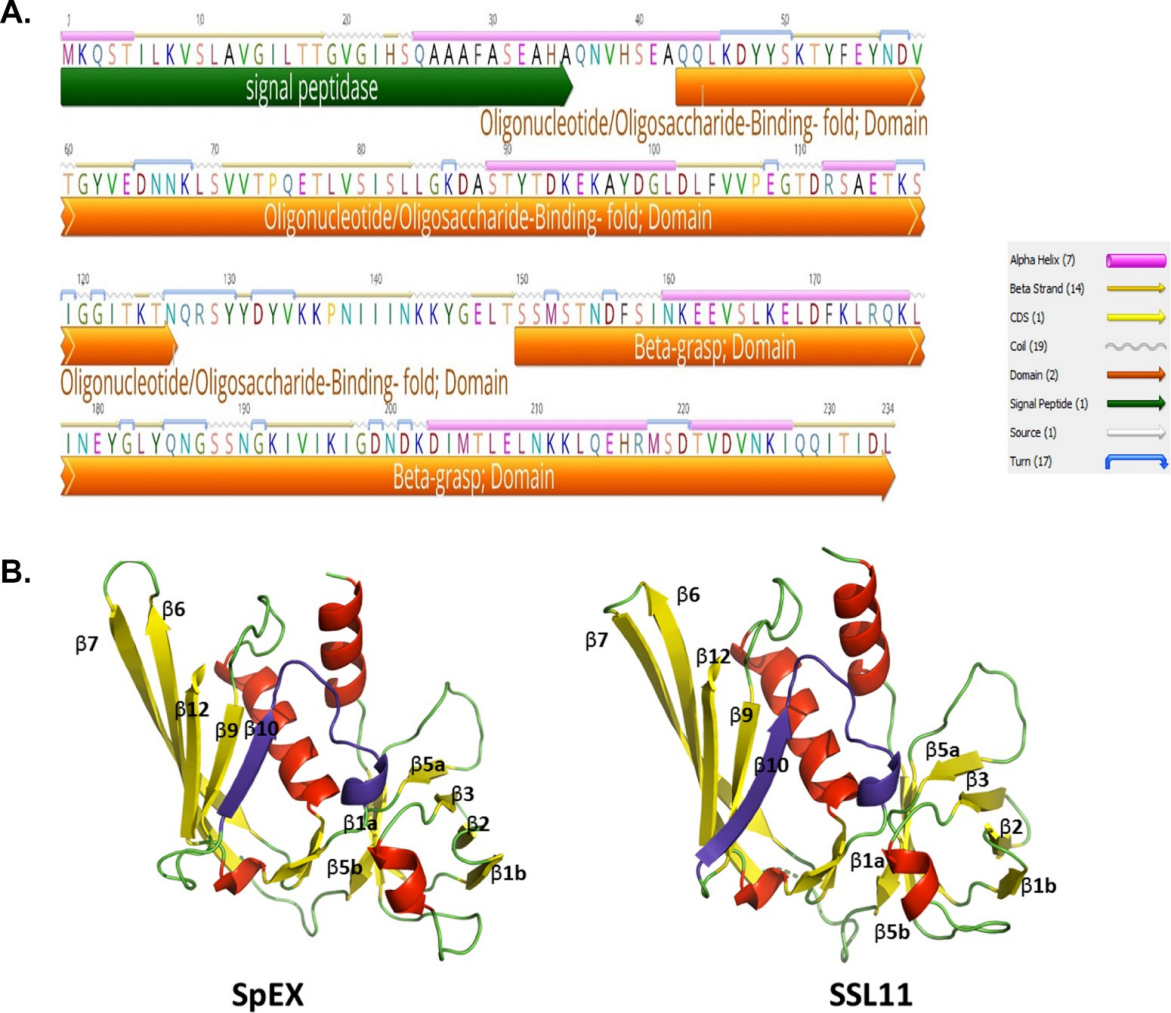
*S*. *pseudintermedius* SpEX structural characteristics. **a.** SpEX of *S*. *pseudintermedius* harbors an N-terminal signal peptide (green arrow) from position 1–35, OB domain (orange arrow) in residues 43–126 and C-terminal β grasp domain in residues 150–234 (orange arrow). **b.** The 3D model of *S*. *pseudintermedius* SpEX and *S*. *aureus* SSL11 shows the N and C terminal domains fold into a five-stranded beta-barrel structure consisting of β1- β5 and β7, β6, β12, β9 and β10. A central helix is orientated diagonally in the center of the field from the top left to the bottom right leaving the N-terminal domain on the left and C-terminal domain on the right. A conserved sialated glycan binding domain forming V-shape depression is highlighted in purple in SpEX and SSL11 3D models.

Analysis of SpEX secondary and tertiary domain structures using Geneious 11.0.3 software [24] supported by a SpEX model developed with the Phyre^2^ web portal showed that *S*. *pseudintermedius* SpEX share fold and domain architecture with *S*. *aureus* SSLs and SEIX (Fig 1A and 1B). The SpEX protein sequence contains a signal peptide sequence from positions 1 through 35 detected using SignalP 4.1 [25]. SpEX has an N-terminal OB domain in residues 43–126 that folds into a five-stranded beta-barrel structure consisting of β-strands (β1- β5) and a C-terminal β grasp motif in residues 150–234 (Fig 1A). This comprises a β sheet formed from β7, β6, β12, β9 and β10 with a characteristic central helix orientated diagonally in the center of the field from the top left to the bottom right leaving the N-terminal domain on the left and C-terminal domain on the right (Fig 1B).

**Fig 2:**
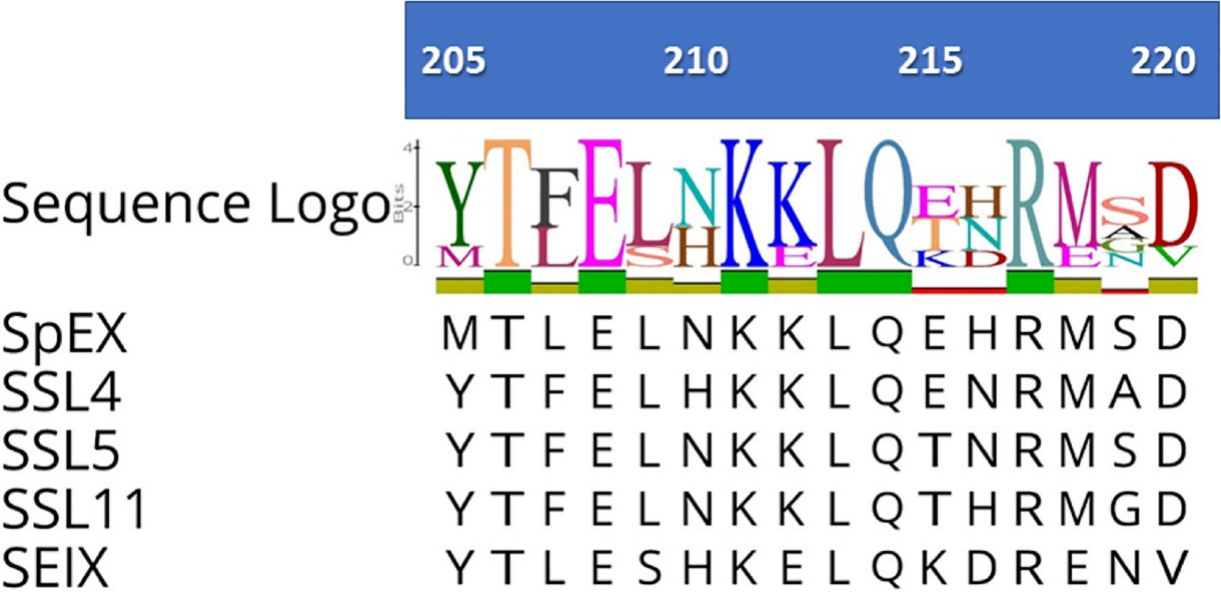
Multiple sequence alignment of SpEX glycan binding domain with *S*. *aureus* SSL and SEIX. Proteins with accession numbers are SSL4 (WP_000705627.1), SSL5 (WP_000784244.1), SSL11 (WP_000769163.1) and SEIX (AEI60186.1). Threonine (T) 206, serine (S) 208, lysine (K) 211, leucine (L) 213 and arginine (R) 217 are identical among all analyzed proteins (five proteins). The numbers refer to the position of the amino acids in full length SpEX protein.

*S*. *aureus* SSL4 (accession number: WP_000705627.1), SSL5 (accession number: WP_000784244.1), SSL11 (accession number: WP_000769163.1) and SEIX (accession number: AEI60186.1) proteins have been shown to bind sialated glycan through a conserved sialated glycan binding domain located at the C-terminal β grasp domain [19, 20, 26, 27]. It has the capacity to influence the host's innate immunity defenses through targeting sialated glycoproteins at the surface of immune cells. Amino acid multiple sequence alignment of SpEX with previously characterized *S*. *aureus* SSL4, SSL5, SSL11 and SEIX proteins showed that *S*. *pseudintermedius* SpEX possesses the conserved sialated glycan binding site of the SSLs located at the C-terminal region and forming the V-shape depression in the 3D model of SpEX (Fig 2).

In *S*. *pseudintermedius* SpEX, we predict aspartic acid (D) 102 and threonine (T) 125 in the OB- fold domain are critical residues for the cytotoxic effect of the protein on immune cells. T 206, serine (S) 208, lysine (K) 211, leucine (L) 213, glutamine (Q) 214, arginine (R) 217 and isoleucine (I) 227 lining the sides of the glycan binding domain were predicted as essential for binding to monocytes, neutrophils and complement. These residues are highly conserved among SSLs and SEIX proteins.

### Design of SpEX with amino acid substitutions

**Fig 3:**
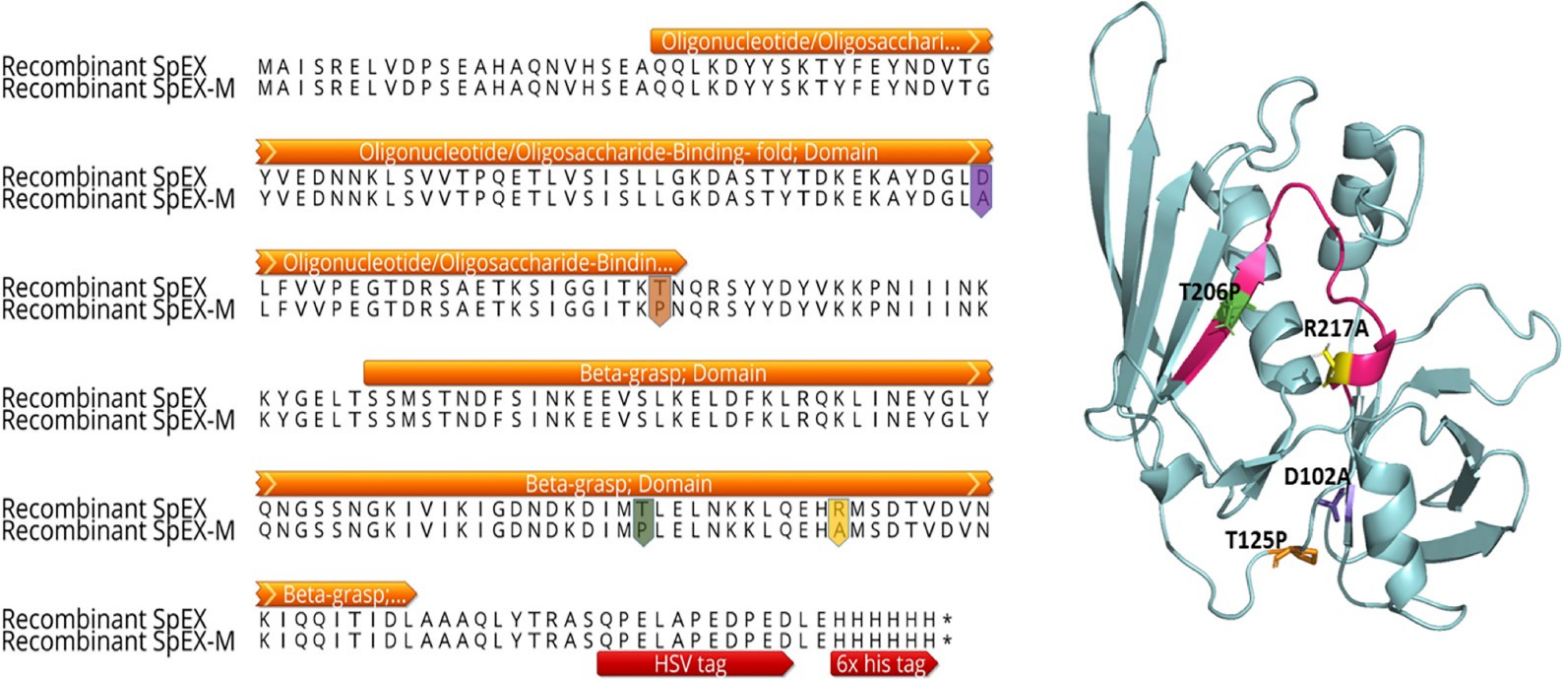
Residues substituted to produce attenuated *S*. *pseudintermedius* SpEX-M. Pairwise amino acid sequence alignment between recombinant SpEX and SpEX-M. Attenuated SpEX-M had substitutions of D102A and T125P (highlighted in purple and orange color, respectively) in the OB-fold domain and T206P and R217A (highlighted in green and yellow color, respectively) in the sialated glycan binding domain. The herpes simplex virus (HSV) tag and 6x his tag were annotated with red arrows whereas the protein domains were annotated with orange arrows.

An attenuated *S*. *pseudintermedius* SpEX (SpEX-M) was designed with (D102A and T125P) in the OB-fold domain and (T206P and R217A) in the sialated glycan binding domain (Fig 3).

To ensure that protein stability and folding were not altered, only amino acids highly conserved within functional domains of *S*. *pseudintermedius* SpEX and *S*. *aureus* SSL4, SSL5, SSL11 and SEIX were selected. Furthermore, the designed SpEX was first modelled in the structure to confirm that its conformation was maintained (Fig 3).

### Cloning, expression, and purification of recombinant *S*. *pseudintermedius* SpEX

**Fig 4:**
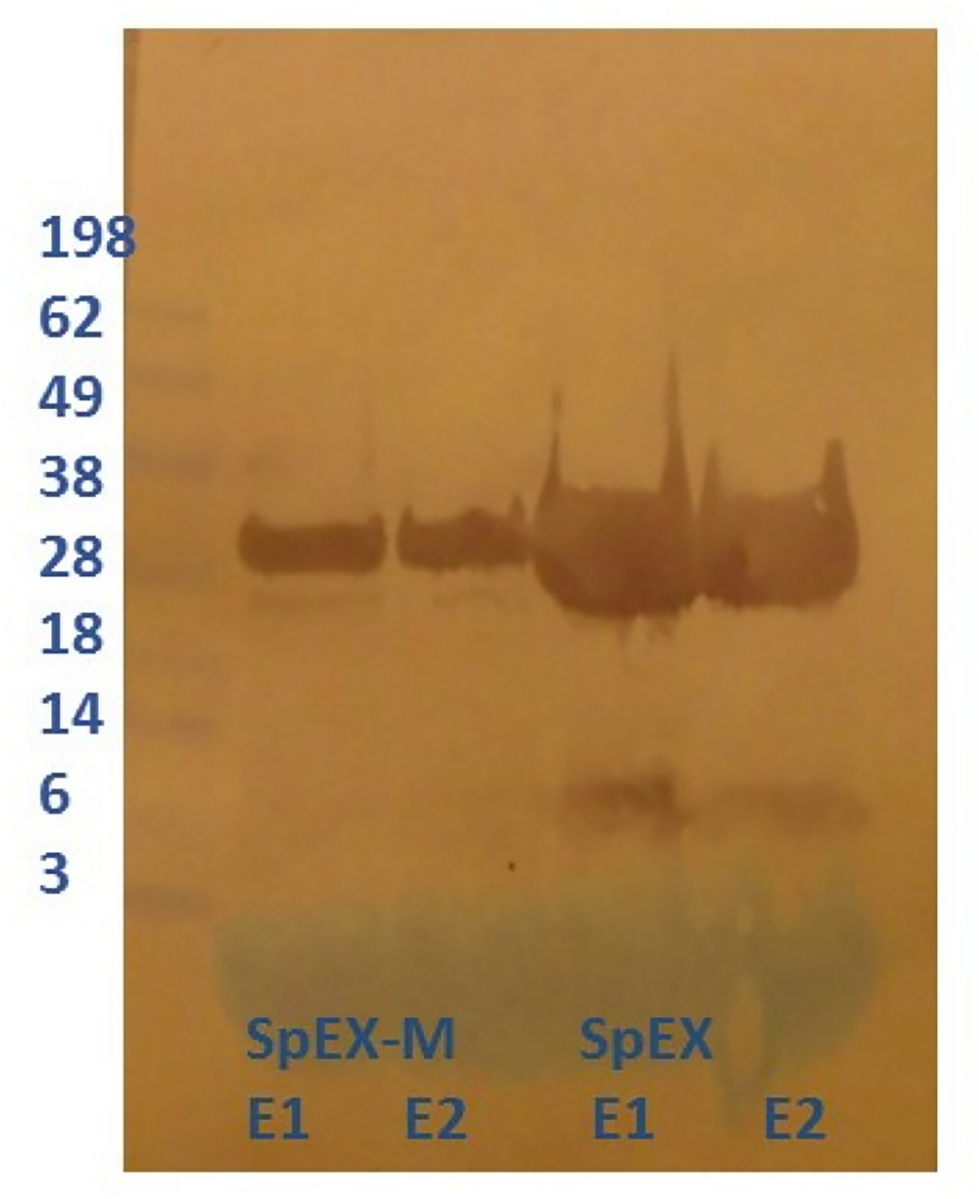
Western blot of recombinant *S*. *pseudintermedius* native and mutant SpEX detected with HRP-conjugated anti-6xhis. The Molecular weights of recombinant SpEX (rSpEX) and rSpEX-M, after expression in Tuner (DE3) pLacI *E*. *coli* induced by Isopropyl β-D-1-thiogalatopyranoside (IPTG), determined in western blots were of the expected sizes (27. 63 and 27.49 kDa, respectively) in the elution fractions (E1 and E2).

Recombinant native and mutant SpEXs with C- terminal 6x histidines (Fig 3) were generated in *E*.*coli* and purified under native conditions using HisPur Ni-NTA affinity chromatography. Both SpEX and SpEX-M expressed well from *E*. *coli* and remained soluble in solution, indicating protein stability with the amino acid substitutions. The molecular weights of SpEX and SpEX-M determined in western blots were of the expected sizes (27. 63 and 27.49 kDa, respectively) (Fig 4).

### SpEX interferes with complement function

**Fig 5:**
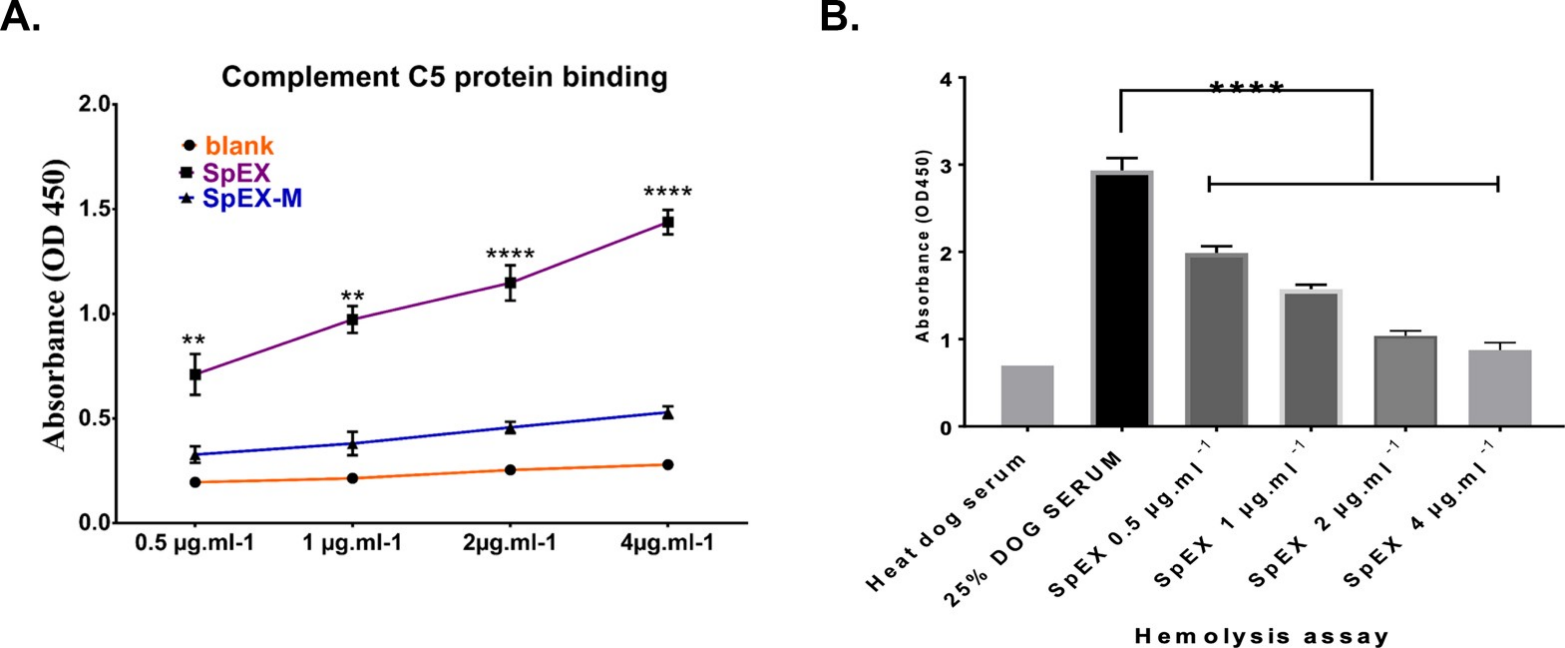
SpEX interferes with complement function. **a.** HRP-conjugated anti-6xhis tag monoclonal antibody was used at a dilution of 1/1000 to detect recombinant SpEX bound to human C5. Recombinant SpEX bound significantly higher to human C5 than recombinant SpEX-M protein (0.5 with P = 0.0171**, 1 μg /ml with P = 0.0027**, 2 and 4 μg /ml with P <0.0001****). These values represent averages from three independent experiments. (*P < 0.05 was considered significant). **b.** Starting at a concentration of 0.5 μg/ml, SpEX significantly reduced the hemolysis of sensitized bovine erythrocytes compared to the positive control with P < 0.0001****. SpEX at a concentration of 4 μg/ml showed no significant difference in hemolysis compared with the negative control. The values represent averages from three independent experiments. (*P < 0.05 was considered significant).

Binding of SpEX to human complement C5 was detected using ELISA, wherein complement component C5 was coated on ELISA plates and binding of recombinant native and attenuated his-tagged SpEX to C5 was measured using horseradish peroxidase (HRP)-conjugated anti-his tag monoclonal antibody. SpEX bound to human C5 significantly higher, in a dose dependent manner (0.5 with P = 0.0171, 1 μg /ml with P = 0.0027, 2 and 4 μg /ml with P <0.0001), than SpEX-M (Fig 5A).

A hemolytic assay was used to evaluate SpEX mediated- inhibition of RBC lysis by complement. SpEX fixed complement and caused inhibition of hemolysis in a concentration dependent manner. At concentrations of 0.5, 1, 2 and 4 μg /ml, SpEX reduced the hemolysis of sensitized bovine erythrocytes compared to the positive control with P < 0.0001. SpEX at a concentration of 4 μg/ml showed no significant difference in hemolysis compared with the negative control (Fig 5B).

### SpEX inhibits neutrophil migration

**Fig 6:**
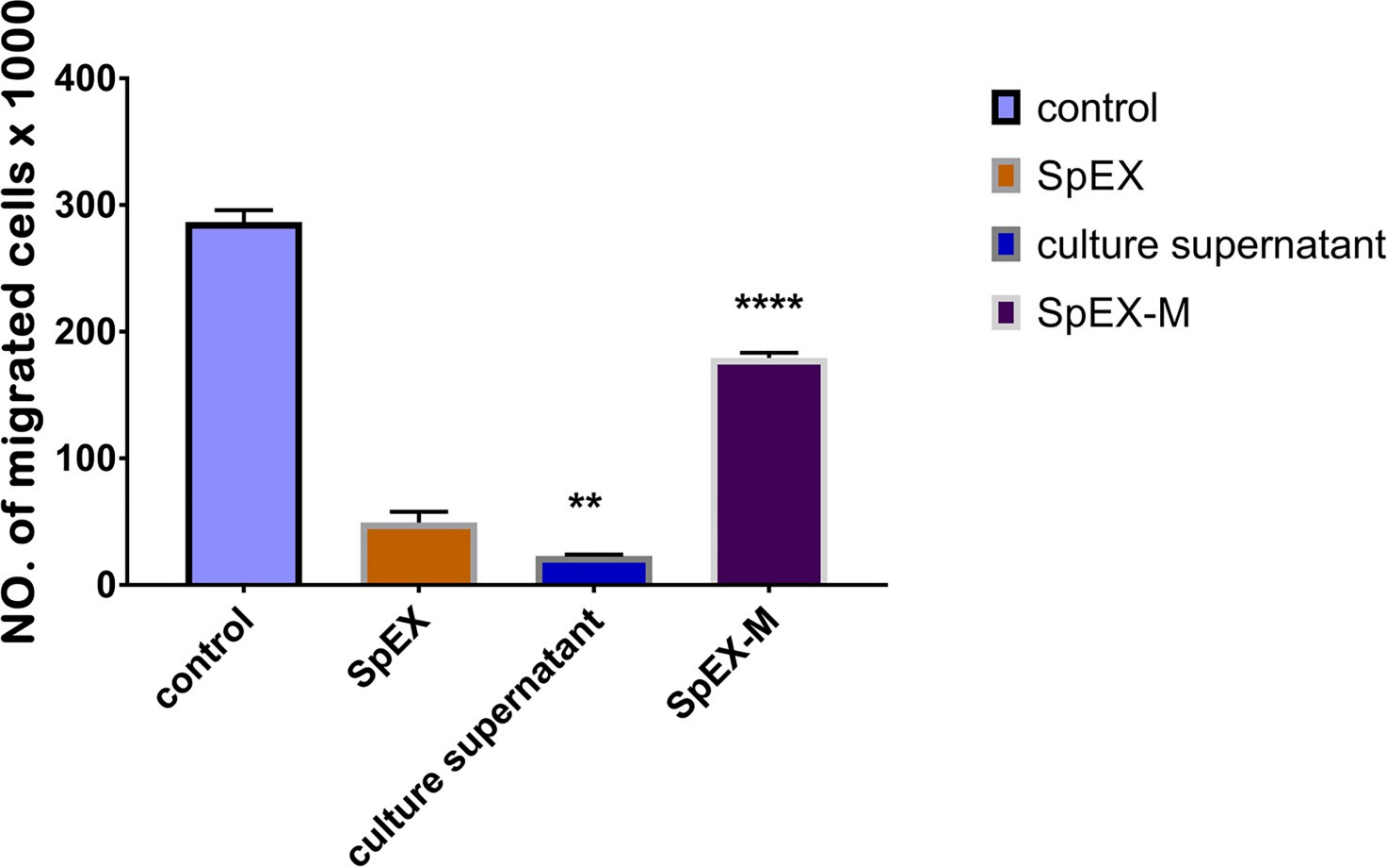
PMN transmigration assay. Recombinant SpEX at a concentration of 0.2 μg /ml significantly inhibited the migration of PMNs induced by fetal bovine serum compared to SpEX-M at the same concentration with P < 0.0001****. The chemotaxis inhibition by culture supernatant of *S*. *pseudintermedius* 06–3228 was significantly higher than SpEX P = 0.0035**. The values represent averages from three independent experiments. (*P < 0.05 was considered significant). Data are plotted on the Y axis X 1000.

A trans-well neutrophil migration assay was used to determine the inhibitory effect of recombinant SpEX on neutrophil migration *in vitro*. Recombinant SpEX at 0.2 μg/ml (at a concentration selected to assure cells remained viable) inhibited the migration of neutrophils induced by fetal bovine serum compared to SpEX-M at 0.2 μg /ml with P < 0.0001 (Fig 6). To validate the results, the viability of neutrophils during experiments was examined to ensure that killing did not occur at the concentration of SpEX used.

### Attenuated SpEX induces a strong antibody response

**Fig 7:**
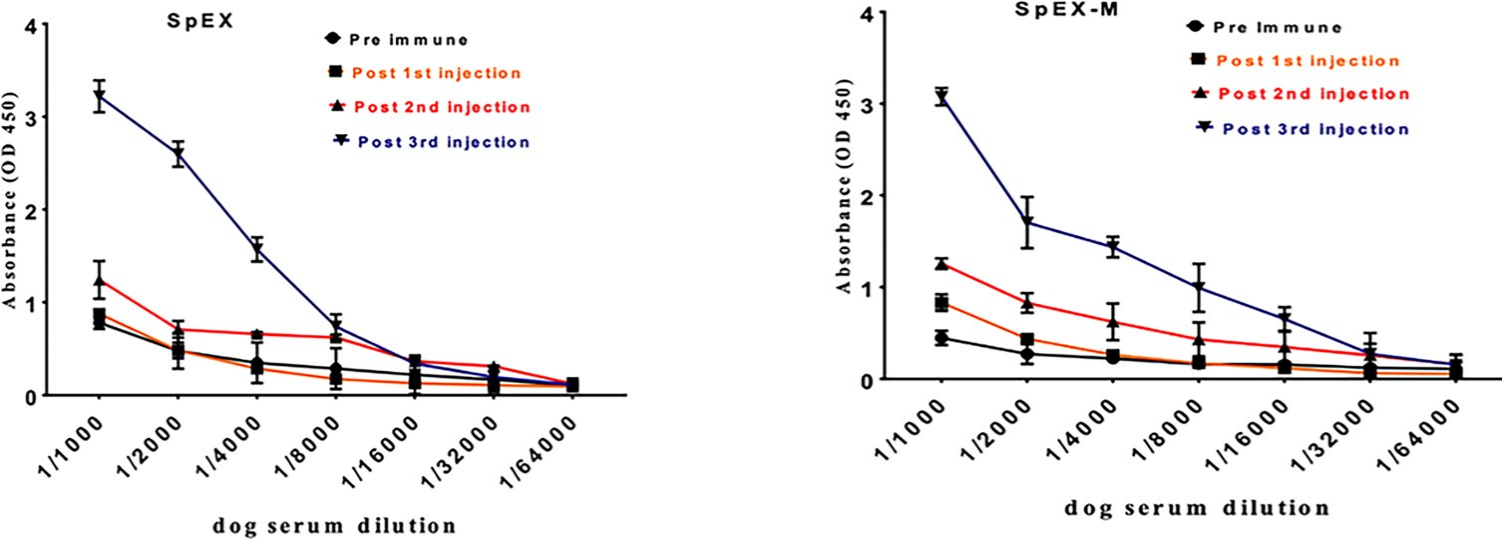
Dogs injected with SpEX-M developed IgG specifically reactive with recombinant native SpEX and SpEX-M. Antibodies against *S*. *pseudintermedius* native SpEX and SpEX-M were measured using an indirect ELISA. Recombinant *S*. *pseudintermedius* SpEX and SpEX-M proteins were coated on ELISA plates, then incubated with two-fold serially diluted serum from dogs vaccinated with the same proteins. High reactivity with SpEX and SpEX-M occurred with sera collected two weeks after the third injections of SpEX-M (P <0.0001) compared to pre-injection sera. The values represent averages from three independent experiments.

Recombinant SpEX-M at 20 μg in 0.5 ml in phosphate buffered saline (PBS) (pH 7.2) was injected into three clinically normal dogs subcutaneously in the lateral thorax. Sera were collected from dogs on days -7, 8, 15 and 29 (relative to SpEX-M injections). IgG reactive with SpEX-M and SpEX was detected by ELISA on day 15 (P < 0.0001) and at a higher level on day 29 (P < 0.0001) compared to pre-injection control sera (Fig 7). Both SpEX and SpEX-M were recognized by dog anti-SpEX-M, confirming that there were no major antigenic differences between native and mutant SpEX.

### SpEX kills canine PMNs and monocytes

**Fig 8:**
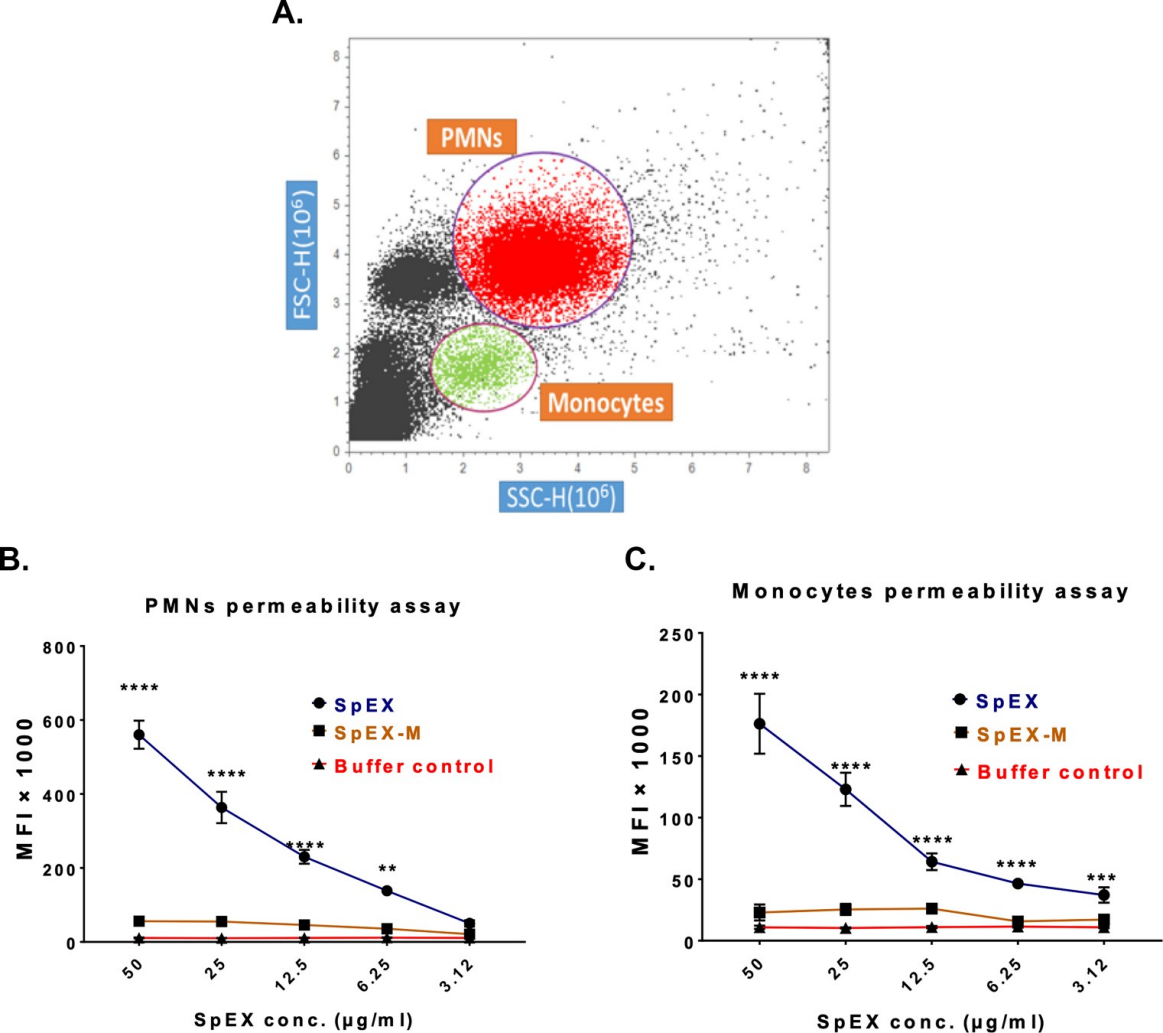
*S*. *pseudintermedius* recombinant SpEX has a cytotoxic effect on canine monocytes and PMNs. **a**. Gating on canine monocytes (green color) and PMNs (red color) was based on side and forward scatter (shown in dot plot). The mean fluorescent intensity (MFI) of the buffer control and SpEX-M relative to SpEX was significantly lower in PMN (P < 0.0001****) and monocyte (P <0.0001****) permeability assays. The values were calculated based on average values from three independent experiments (*P < 0.05 was considered significant, **** P <0.0001, **** P = 0.0043).

Canine PMNs and monocytes harvested from canine blood were highly susceptible to SpEX with cell permeability induced within 30 minutes in a concentration dependent manner (50, 25, 12.5, 6.25, 3.12 μg SpEX/ml in PBS, pH 7.2) and by a 1:2 dilution of *S*. *pseudintermedius* strain 06–3228 culture supernatant (Fig 8). SpEX-M showed a diminished effect on cell permeability of canine PMNs (P = 0.0052) (Fig 8) and monocytes compared to SpEX (P < 0.0001) (Fig 8). At SpEX concentrations of 50, 25, 12.5, 6.25 and 3.12 μg/ml, monocyte cell permeability was significantly different from that of SpEX-M and the negative control (P < 0.0001) (Fig 8). However, SpEX at concentrations of 50, 25, 12.5 and 6.25 μg/ml, had a significant effect on PMN cell permeability compared to SpEX-M and negative control (P < 0.0001) (Fig 8).

### Canine anti-SpEX-M reduced the effects of SpEX on PMNs and monocytes *in vitro*

**Fig 9:**
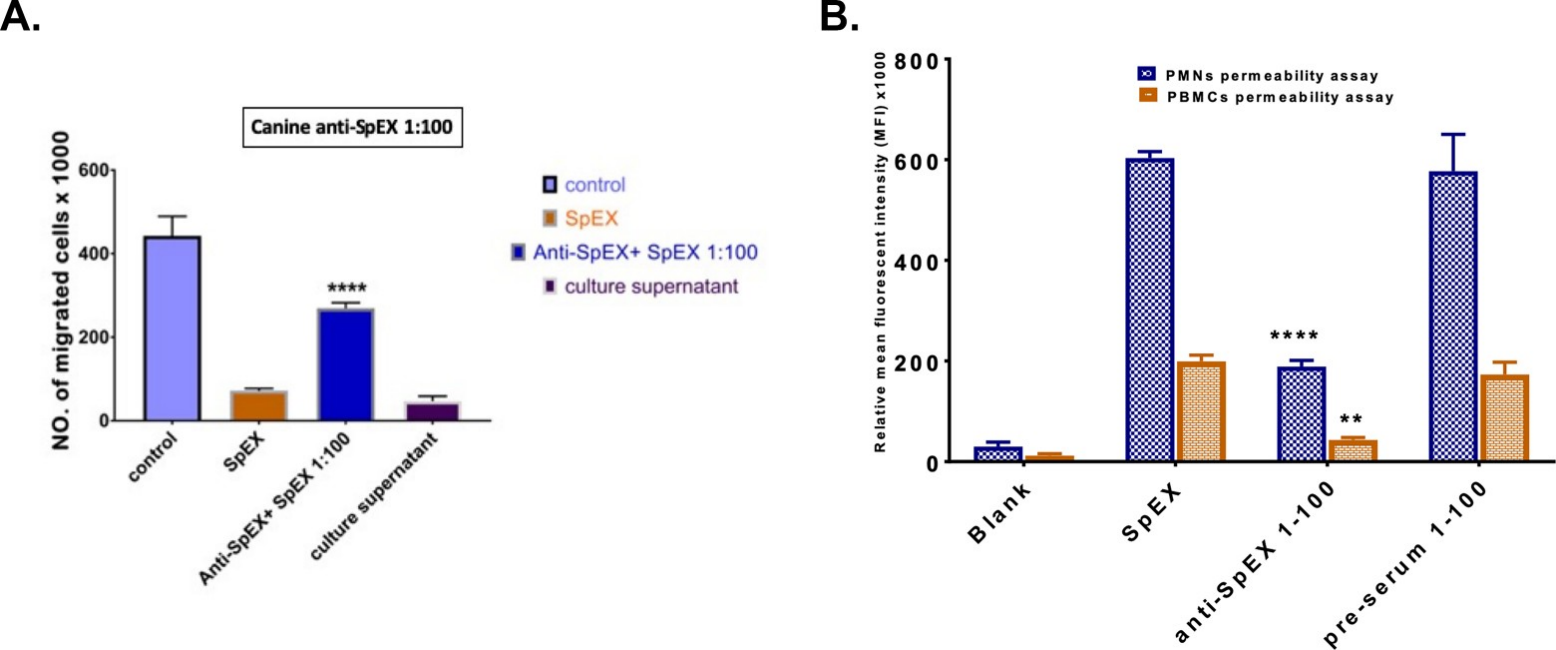
Canine anti-SpEX-M neutralized SpEX. **a.** Canine anti-SpEX, collected after three injections, at a dilution of 1:100 in PBS (pH 7.2), preincubated with recombinant SpEX, significantly diminished the chemotaxis inhibition of SpEX with P < 0.0001****. **b.** Pre-incubation of canine anti-SpEX diluted 1:100 with SpEX resulted in a significant reduction in MFI in permeability assays as compared with that of SpEX treatment alone. There was no significant difference between SpEX and SpEX treated with pre-serum. The MFI of SpEX pre-incubated with anti-SpEX 1:100 relative to SpEX was significantly lower in PMN (P < 0.0001****) and monocyte (P = 0.012**) permeability assays. The values calculated were based on average values from three independent experiments (*P < 0.05 was considered significant).

Canine anti-SpEX-M collected after 3rd injection, at a dilution of 1:100 in PBS (pH 7.2), preincubated with recombinant SpEX at a concentration of 0.2 μg /ml, significantly reduced the inhibitory effect of rSpEX on leukocyte chemotaxis compared to serum collected from dogs before SpEX-M injection and control serum with P < 0.0001 (Fig 9A).

In a measure of the protective effect of canine anti-SpEX-M against the native protein, rSpEX (3.1 μg per ml of PBS) treated with anti-SpEX-M at dilution 1:100 significantly reduced cell permeability represented by mean fluorescent intensity (MFI) as compared with that of SpEX treatment alone (Fig 9B).

## Discussion

Using mass spectrometry referenced to genomic data, it was possible to identify a new *S*. *pseudintermedius* exotoxin, SpEX, in *S*. *pseudintermedius*. SpEX secretion was confirmed in three *S*. *pseudintermedius* strains representing the three clonal complexes that predominate in the United States [5]. SpEX from the most commonly isolated clonal complex, CC68, was used in this study. However, SpEX is highly conserved among all three clonal complexes. Although its sequence is unique, this protein has a typical SSL tertiary structure, shared by *S*. *aureus* SSLs and SEIX, consisting of an N-terminal OB-fold domain that folds into a five-stranded β-barrel and a C-terminal β-grasp domain. However, SpEX, in addition to sharing the chemotaxis and complement inhibitory properties of SSLs, has a cytotoxic effect against monocytes and PMNs. These functions likely promote bacterial survival in their hosts and increase the likelihood of transmission. The attenuated SpEX remained soluble following expression from *E*. *coli* and both SpEX and SpEX-M were recognized by dog anti-SpEX-M. Thus, SpEX-M with amino acid substitutions in its predicted functional domains did not induce significant antigenic or conformational changes in the protein, however it showed diminished immune evasive properties compared to SpEX.

Antibody-mediated toxin neutralization may provide a strategy for immunotherapeutic treatment and/or prevention of current and recurrent infection [28, 29]. Canine antibodies to SpEX neutralize and diminish its chemotactic inhibitory and cytotoxic effects on PMNs and monocytes highlighting the potential value of this protein as a component in a vaccine to prevent or treat *S*. *pseudintermedius* infections.

The increased prevalence of multidrug resistant *S*. *pseudintermedius leaves few* options for antimicrobial therapy [5]. Therefore, the development of novel strategies to treat this pathogen are a research priority [5, 30]. One alternative approach is the development of a vaccine that can confer protection or provide effective therapy. However, vaccines in *S*. *aureus* clinical trials have thus far failed to show efficient protection [31]. Previous studies [30, 32–34] concluded that multicomponent vaccines containing a cocktail of staphylococcal antigens would likely work best in preventing infections caused by *S*. *aureus*. We propose that a potent and effective vaccine against *S*. *pseudintermedius* would involve immunogenic targets, secreted and/or accessible on the surface of the bacterium, conserved among prevalent strains and that play important roles in virulence, such as immune evasion. Understanding *S*. *pseudintermedius* protein function, surface accessibility and epitope conservation is crucial for vaccine development against infections caused by this pathogen.

In conclusion, we describe a new exotoxin produced by *S*. *pseudintermedius* that binds to C5, inhibits complement activation and permeabilizes leukocytes. A mutant version of the protein has reduced cytotoxic effects on dog PMNs and monocytes *in vitro*. Canine anti-SpEX produced against an attenuated form of the protein reduced its chemotaxis inhibition. Therefore, these mutant proteins may serve as important components of a multivalent vaccine for prophylaxis of *S*. *pseudintermedius* infections. By neutralizing extracellular toxins responsible for host tissue destruction and immunosuppression, such a vaccine may help the host immune system control infections.

Future studies might examine potential interactions with TLR as well as regulation of *SpEX* expression and the effects of quorum sensing and biofilm on production of the protein to better understand its role in pathogenesis. In addition, orthologs of SpEX produced by other members of the *Staphylococcus intermedius* group should be studied to determine their role in the virulence of these species.

## Materials and methods

### Ethics statement

Experimental protocols were reviewed and approved by the University of Tennessee Institutional Animal Care and Use Committee (IACUC) including 2474–0716 for samples of dog blood and 2572–1217 for producing antibodies in dogs with recombinant protein.

### Bacterial strains and plasmids

Log phase bacterial cultures of *S*.*pseudintermedius* strains (shown with their sequence types) 06–3228 (ST68), 08–1661 (ST71) and NA45 (ST84) were analyzed by mass spectrometry. These strains are representative of the major *S*. *pseudintermedius* genotypes (clonal complexes) most commonly isolated from canine infections in the United States. A plasmid construct containing a mutated, synthetic *S*. *pseudintermedius spEX* (designed as described below) with BamHI/NotI cloning sites, was obtained commercially (Genscript Piscataway, NJ USA) (Table 1).

**Table 1:** Bacteria, plasmids and competent cells used in this study.

Plasmid/ Bacteria	Characteristics	Source
**pUC19-SpEX-M construct**	Cloning plasmid containing synthetic, attenuated *S*. *pseudintermedius* SpEX (717 bp) inserted into MCS of pUC19 between BamHI and NotI restriction sites	Genscript Piscataway, NJ USA
**pETBlue-2**	Plasmid for T7 promoter based expression of recombinant proteins with blue/white screening and C-terminal HSVTag and HisTag sequences	Novagen, Madison, WI
**pETBlue-2 SpEX construct**	Plasmid containing synthetic *S*. *pseudintermedius* SpEX (716 bp) inserted into MCS of pETBlue-2 between BamHI and NotI restriction sites and C-terminal HisTag sequences	This study
**pETBlue-2 SpEX-M construct**	Plasmid containing synthetic *S*. *pseudintermedius* SpEX-M (717 bp) inserted into MCS of pETBlue-2 between BamHI and NotI restriction sites and C-terminal HisTag sequences	This study
**DH5α *E*.*coli***	Competent cells used for cloning pETBlue-2 constructs. Genotype: F^-^ Φ80*lac*ZΔM15 Δ (*lac*ZYA-*arg*F) U169 *rec*A1 *end*A1 *hsd*R17 (r_k_^-^, m_k_^+^) *pho*A *sup*E44 *thi*-1 *gyr*A96 *rel*A1 λ^-^.	Novagen, Madison, WI
**Tuner(DE3) pLacI *E*.*coli***	lacZY deletion mutants of BL21 and a lysogen of λDE3 that carries a chromosomal copy of the T7 RNA polymerase gene under control of the lacUV5 promoter.	Novagen, Madison, WI
***S*. *pseudintermedius* 06–3228**	Strain representing the most common ST in the United States (ST68)	*
***S*. *pseudintermedius* 08–1661**	Strain representing the most common ST in Europe and among the three most common in the United States (ST71)	*
***S*. *pseudintermedius* NA45**	Strain representing the most common ST in Asia and among the three most common in the United States (ST84)	**

*University of Tennessee, College of Veterinary Medicine Bacteriology Laboratory.

** A gift of Faye Hartmann of the University of Wisconsin, School of Veterinary Medicine.

### Media and growth conditions

Bacterial colonies grown on blood agar plates were inoculated into 5ml of trypticase soy broth (TSB) (BD Biosciences, San Jose, CA) at 37°C with shaking at 225 rpm. For log-phase bacterial cultures, bacteria were grown until an optical density (OD _600_) of 0.4–0.6 was reached.

### LC-MS/MS analysis of *S*. *pseudintermedius* supernatant

Log phase bacterial cultures of 06–3228, 08–1661 and NA45 were centrifuged at 10,000g for 30 minutes at 4°C and the supernatants were collected and passed through 0.45μm filters (Whatman, GE Healthcare Lifesciences, Pittsburgh, PA). The filtrates were concentrated using Amicon ^Ultra^-4 centrifugal filters (EMD Millipore Corp., Billerica, MA) and stored at -20°C until further analysis. Samples interrogating *S*. *pseudintermedius* supernatant were prepared for shotgun LC-MS/MS analysis. Trypticase soy broth (media alone) and bacterial culture supernatant passed through control Sepahrose beads (without IgG) served as controls. Peptides were separated and analyzed with a 2-step MudPIT LC-MS/MS protocol (salt cuts of 50mM and 50mM ammonium acetate) over a 4-hr period then measured with a hybrid LTQ XL-Orbitrap (Thermo Scientific, Waltham, MA) mass spectrometer (MS). The percent coverage of detected proteins was calculated as previously described [35].

### Bioinformatics analysis

Multiple sequence alignment (MSA) of SpEX proteins from a total of 123 *S*. *pseudintermedius* isolates available in the Genbank database and others sequenced previously in our lab [36–38] was performed using Geneious 11.0.3 software [24]. Protein Homology/analogY Recognition Engine V 2.0 (Phyre^2^) was used to develop a *S*. *pseudintermedius* SpEX model (http://www.sbg.bio.ic.ac.uk/phyre^2^) with alignment coverage, identity percent and confidence set to 82%, 100% and 40% respectively [39]. The 3DLigandSite online tool (http://www.sbg.bio.ic.ac.uk/3dligandsite/) was used for binding site prediction [40].

The *S*. *pseudintermedius* SpEX sequence was aligned to *S*. *aureus* SSL4, SSL5, SSL11 and SEIX to identify the shared protein domain and secondary structures. We designed a full-length, attenuated *S*. *pseudintermedius* SpEX construct (SpEX-M), with the following amino acid substitutions: D102A, T125P, T206P and R217A.

### Polymerase chain reaction (PCR) amplification of *SpEX*

DNA was extracted using a MO BIO UltraClean Microbial DNA Isolation Kit (QIAGEN Inc.) according to the manufacturer’s instructions. Oligonucleotide primers (Integrated DNA Technology, Coralville, USA) (Table 2) were designed using the PrimerQuest Tool (https://www.idtdna.com/Primerquest/Home/Index) based on the genomic sequence of *S*. *pseudintermedius* strain 06–3228 [41].

**Table 2:** Primers used to amplify recombinant wild- type and attenuated *spEX* from *Staphylococcus pseudintermedius* and pUC19-*spEX-*M plasmid. NotI and BamHI restriction sites are bold and underlined, nucleotide sequences belonging to SpEX and SpEX-M are show in italics, the primer binding sites are in brackets and sequences outside SpEX and SpEX-M (GCATGA) were used to flank NotI and BamHI recognition sites.

Primer	Sequence
**SpEX forward**	GCATGAGGATCCA[*AGCGAAGCACATGCCCA*]
**SpEX reverse**	GCATGAGCGGCCGC[*CAGATCTATCGTAATTTGTTGGA*]
**SpEX-M forward**	GCATGA[GGATCCA*ATGAAACAGAGCACCATTCT*]
**SpEX-M reverse**	GCATGA[GCGGCCGC*CAGGTCGATG*]

The native *SpEX* open reading frame (ORF) (705bp) excluding N-terminal signal peptide was amplified from *S*. *pseudintermedius* strain 06–3228 genomic DNA and the mutant *SpEX* was amplified from a pUC19-*spEX-*M plasmid (Genscript Piscataway, NJ USA) (Table 1) using taq polymerase (rTaq, Takara).

### Cloning, expression, and purification of recombinant native and attenuated SpEX

*S*. *pseudintermedius* native and mutant *SpEX* PCR products were cloned using NotI and BamHI digested pETBlue-2 (Novagen,) (Table 1). The plasmid constructs were transformed into cloning host DH5-alpha *E*. *coli* chemically-competent cells, (Table 1) (New England BioLabs Inc.,) before being transformed into expression host, Tuner (DE3) pLacI *E*. *coli* chemically-competent cells (Table 1) (Novagen,) by heat shock.

Recombinant *S*. *pseudintermedius* native and mutant SpEX expression were performed from *E*. *coli* pETBlue-2 constructs as previously described [4] using LB broth containing 50μg/ml ampicillin and 20μg/ml chloramphenicol as protein expression media. Protein expression was induced by addition of 1 mM Isopropyl β-D-1-thiogalatopyranoside (IPTG). Protein extraction was performed using BugBuster reagent (Novagen) and 100X protease inhibitor (Cocktail Set III, EDTA-Free Calbiochem,). Recombinant proteins were purified using affinity purification (HisPur Ni-NTA Spin Purification Kit, Thermo Scientific). Protein concentrations were determined using a bicinchoninic acid (BCA) assay (Thermo Scientific).

### SDS-PAGE and western blot

SDS-PAGE in 4–12% polyacrylamide gels (Invitrogen) was used to measure the molecular weight of expressed recombinant proteins. The resolved protein samples were blotted onto nitrocellulose membranes (Thermo Scientific). Then the blots were incubated in 5% (wt/vol) nonfat dried milk powder in 0.05% polyethylene glycol sorbitan monolaurate (Tween 20) containing phosphate buffered saline (PBS-T) overnight at 4°C. Horseradish peroxidase (HRP)-conjugated anti-6xhis tag monoclonal antibody (Thermo Scientific) diluted 1:2,000 in 0.05% PBS-T was added to the blocked membranes and incubated for 1 h with 225 rpm shaking at room temperature. After five washes with 0.05% PBS-T, bound antibodies were detected using 1-Stepchloronaphthol substrate solution (Thermo Scientific).

### Preparation of canine anti- *S*. *pseudintermedius* SpEX

The endotoxin concentration in purified recombinant SpEX-M was measured using a ToxinSensor Chromogenic LAL Endotoxin Assay Kit (Genscript). Recombinant SpEX-M at 20 μg was diluted in 0.5 cc in phosphate buffered saline (PBS) (pH 7.2) and injected into three clinically normal dogs subcutaneously in the lateral thorax. Three injections were given 7 days apart with a control dog receiving PBS (pH 7.2) only. Blood was drawn on days -7, 8, 15 and 29 (relative to injections) and serum separated.

### Enzyme-linked immunosorbent assay

Recombinant SpEX and SpEX-M were coated separately onto ELISA plates (Corning) at 2μg/ml in PBS. The plates were washed with 0.05% PBS-T and incubated with two-fold serial dilutions of serum from dogs (injected with recombinant proteins) for 1 h at 37°C, then bound IgG was detected using HRP-conjugated goat anti-dog IgG-heavy and light chain (Bethyl Laboratories, Inc.). ELISA assay plates were washed three times with PBS-T between all incubations, bound antibodies were detected using TMB substrate (Thermo Scientific), and reactions were stopped with 0.18 M sulphuric acid and optical density read at 450 nm on a plate reader (Bio TEK, EL800). The experiment was repeated a minimum of three times and a p-value of <0.05 was considered significant for all the experiments unless otherwise stated.

### Trans-well assay for neutrophil transmigration

Canine blood was collected from healthy dogs using a sterile blood collection system with EDTA anticoagulant (BD Vacutainer). Canine neutrophils were isolated according to O’Donnell [42], washed several times, and re-suspended in pre-warmed 1ml RPMI medium.

A neutrophil transmigration assay protocol was used as previously described [43] with modifications. The assay was performed in 24-well plates with polycarbonate membrane (3.0 μM pore size and 6.5 mm well diameter) chambers (Corning Incorporated). Six hundred microliters of Dulbecco's Modified Eagle Medium (DMEM) with 10% of fetal bovine serum, as a PMN chemoattractant, was placed in each bottom well. Subsequently, SpEX and SpEX-M treated canine neutrophils (1×10^6^), were added to the top chamber and incubated at room temperature for 6 h. DMEM medium was used as a negative control. Neutrophil viability and migration to the bottom chamber was quantified using a Countess II FL Automated Cell Counter (Thermo Scientific). In order to measure the protective effect of anti-SpEX on neutrophil transmigration, recombinant SpEX was incubated with canine anti-SpEX at a dilution of 1:100 for 30 min at 37°C, then tested with the PMN transmigration assay.

### Complement C5 binding assay

Human complement component C5 (Sigma-Aldrich) was coated onto ELISA plates (Corning) at 2μg/ml in PBS. Recombinant SpEX and SpEX-M were added at 0.5, 1, 2 and 4 μg/ml in PBS (pH 7.2) for 1 h at 37°C, then bound recombinant proteins were detected using (HRP)-conjugated anti-6xhis tag monoclonal antibody (Thermo Scientific) at a dilution of 1:1000 in PBS-T (pH 7.2). Bound antibodies were detected as described above. The experiment was repeated a minimum of three times and a p-value of <0.05 was considered significant for all the experiments unless otherwise stated.

### Complement mediated hemolysis assay

A hemolysis assay was performed to determine the ability of recombinant SpEX to inhibit complement function. Bovine erythrocytes, collected in EDTA from a clinically normal cattle, were washed in PBS and sensitized to complement by incubation with rabbit IgG fraction anti-bovine red blood cells (ICN Cappel) diluted 1:25, for 30 min at 37°C with gentle mixing. Dog serum diluted 1:4 was pre-incubated with 0.5, 1, 2 and 4 μg/ml of recombinant native SpEX for 30 min at 37°C with gentle shaking (100 rpm). One hundred microliters of sensitized bovine RBCs were added to all samples including positive and negative controls and incubated under the same conditions for an additional 30 min. After centrifugation at 4200 *×ɡ* for 5 min, the absorbance of the supernatant was measured at 450 nm. Heat-inactivated serum mixed with PBS (pH 7.2) was used as a negative control and normal dog serum, diluted 1:4 was used as a positive control.

### PMN and monocyte permeability assay

PMNs and monocytes were separated as previously described. PMNs and monocytes were incubated with two- fold serial dilutions of recombinant proteins (SpEX or SpEX-M) from concentrations of 50 μg to 1.6 μg in a volume of 500 μl of RPMI medium supplemented with 10% fetal bovine serum in an incubator with 5% CO_2_ for 30 min. The supernatant of *S*. *pseudintermedius* strain 06–3228 was harvested at log phase to test the toxic effect of secreted, native SpEX. PMNs and monocytes were stained with 1 μl Sytox green (Life Technologies, Inc.) for 30 min, then washed twice with PBS (pH 7.2) and analyzed using a flow cytometer (Attune acoustic focusing cytometer) by gating separately on PMNs and monocytes.

To evaluate the protective effect of canine anti-SpEX-M on PMNs and monocytes, recombinant *S*. *pseudintermedius* SpEX, at concentration of 3.1 μg diluted in 1ml of PBS (pH 7.2) was incubated for 30 minutes at 37°C with serum from SpEX-M injected dogs. The cell death cut-off used for flow cytometry analysis was established using leukocytes incubated without SpEX. Mean fluorescent intensity was measured from all gated cells.

### Statistical analysis

Each experiment was repeated at least three times with a minimum of duplicate samples. Data analysis was conducted for each response variable using ANOVA methods, with treatment and dose as the independent variables. Diagnostic analysis was performed to check the model assumptions for normality and equal variance. Multiple comparisons were made with tukey's adjust. Significance was identified at p<0.05. All analysis was conducted in SAS 9.4 for Windows x64 from SAS Institute (Cary, NC) and some graphical outputs were generated by GraphPad Prism software (Version 7, GraphPad Software Inc.).

## Supporting information

S1 Fig Fig 5A Complement C5 protein binding. (XLSX)

S2 Fig Fig 5B SpEX inhibited hemolysis. (XLSX)

S3 Fig Fig 6 Transmigration assay data. (XLSX)

S4 Fig Fig 7 SpEX ELISA data. (XLSX)

S5 Fig Fig 7 SpEX-M ELISA data. (XLSX)

S6 Fig Fig 8B Monocyte permeability. (XLSX)

S7 Fig Fig 8B PMN permeability. (XLSX)

S8 Fig Fig 9A Canine anti-SpEX chemotaxis inhibition. (XLSX)

S9 Fig Fig 9B Canine anti-SpEX neutralization of toxicity. (XLSX)
